# Experiences and needs of residents with dementia in relocating to an innovative living arrangement within long-term care: A qualitative study

**DOI:** 10.1177/14713012241311433

**Published:** 2024-12-21

**Authors:** Mara Brouwers, Elleke GM Landeweer, Bram de Boer, Wim G Groen, Miranda C Schreuder, Hilde Verbeek, RELOCARE consortium

**Affiliations:** Department of Health Services Research, CAPHRI Care and Public Health Research Institute, 5211 Maastricht University, the Netherlands; Living Lab in Ageing and Long-Term Care, the Netherlands; Department of Primary and Long-term Care, University Medical Center Groningen, 3647 University of Groningen, the Netherlands; Department of Care Ethics, University of Humanistic Studies, the Netherlands; Department of Health Services Research, CAPHRI Care and Public Health Research Institute, 5211 Maastricht University, the Netherlands; Living Lab in Ageing and Long-Term Care, the Netherlands; Department of Medicine for Older People, Amsterdam UMC, Vrije Universiteit Amsterdam, the Netherlands; Amsterdam Public Health Research Institute, Aging & Later Life, Amsterdam, the Netherlands; Amsterdam Movement Sciences, Ageing & Vitality, Rehabilitation & Development, Amsterdam, the Netherlands; Department of Primary and Long-term Care, University Medical Center Groningen, 3647 University of Groningen, the Netherlands; Department of Health Services Research, CAPHRI Care and Public Health Research Institute, 5211 Maastricht University, the Netherlands; Living Lab in Ageing and Long-Term Care, the Netherlands

**Keywords:** dementia, innovative housing with care, relocations, care environment, nursing

## Abstract

During the last decade, an increasing number of care organizations have chosen to rebuild or build a new care facility to provide better person-environments for residents with dementia. This has inevitably led to an increase in relocations. This study investigated how residents with dementia experienced a relocation from a regular nursing home to an innovative living arrangement. A qualitative study was performed, using semi-structured interviews and observations. Two nursing homes offering 24 h care to residents with psychogeriatric symptoms that planned a relocation to an innovative living arrangement were selected. Sixteen residents were included. Five themes from the data described what was of importance to residents when moving, including (1) the physical environment of the new location, (2) the belongings of residents, (3) feeling at home, (4) the importance of social contact when relocating, and (5) the need to be engaged in daily life. This study found that the residents were not actively involved in the relocation process, despite the clear desire they expressed to be involved and of importance. As the residents with dementia were able to express what was important to them in this study, relocation processes should focus more on involving such residents and incorporating them within relocation processes.

## Background

Relocating within long-term care is often seen as an impactful life event for older adults with dementia (De Boer et al., 2022). Relocating can lead to an increase in morbidity and mortality and a decline in physical and psychological functioning (Ryman et al., 2019). Residents often regard a relocation as uncontrollable and involving feelings of uncertainty (Falk et al., 2011). Furthermore, studies show that residents can experience a variety of negative emotions, like feelings of loss, deception, fear, frustration, and anger, when they have to relocate involuntarily to a new facility (Capezuti et al., 2006; Leyland et al., 2016). Although some studies have focused on the experiences of residents when relocating within long-term care, these are scarce (Weaver et al., 2020). Studies that have focused on the experiences of residents included residents without, or with minor, cognitive impairments.

Research suggests that both residents with dementia like those without dementia can experience the same amount of relocation stress, meaning that both groups are equally susceptible to, for example, an increase in depressive symptoms, or a decline in physical functioning (Costlow & Parmelee, 2020; Mirotznik & Kamp, 2000). It is noteworthy that the perspectives of residents with dementia have been under-researched, despite the fact that actively involving residents in relocation processes might reduce negative outcomes and possibly foster positive results (Bekhet et al., 2011; Holder & Jolley, 2012; Jolley et al., 2011; Leyland et al., 2016; Weaver et al., 2020). Research shows that individuals with dementia have a strong desire to remain central in decision-making. Not involving them or asking for their opinion led to feelings of marginalization and exclusion (Fetherstonhaugh et al., 2013). It is therefore important to understand the precise experiences and needs of residents.

In recent years, a culture change has been taking place with the aim to de-institutionalize long-term care and improve quality of life and care (Koren, 2010; Zimmerman et al., 2014). As a result, more and more care organizations have chosen to reconstruct old facilities or build new facilities in order to create environments better suited to provide person-centred care to individuals with dementia. This includes a specifically and sometimes radically redesigned physical environment but may also encompass revamping social and psychological aspects, such as promoting autonomy, self-identity, control, choice, and a socially supportive environment (Cutchin et al., 2003; Moore, 2000). These new locations are often referred to as innovative living arrangements (Brouwers et al., 2023). The development of these arrangements has inevitably led to an increase in relocations. How the new environment is shaped, however, affects the individual experiences of residents, which can, in turn, affect their well-being and adjustment following a relocation (Bekhet et al., 2011; Ibrahim et al., 2022; Nowlan et al., 2015). For example, the notion of ‘feeling at home’, which is often a central value of innovative living arrangements, is an important aspect in evaluating relocations, and can be seen as a marker of a successful relocation process (Cutchin et al., 2003). In order to create a place that enables at-homeness, knowing what matters to an individual is key (Cater et al., 2022; Molony et al., 2011; van Hoof et al., 2016). Therefore, gaining insight into the subjective experiences of residents during relocations is crucial to help optimize relocation processes and maintain a sense of belonging, meaningfulness, security, and autonomy.

When relocating to innovative living arrangements, residents with dementia encounter several opportunities and challenges, ranging from the promise of enhanced person-centred care to the disruption of established routines and social networks. Yet, despite the growing prevalence of such settings, the experiences of residents with dementia navigating relocations in general, and in particular relocations to innovative living arrangements, remain an under-researched topic. Although including people with dementia as participants in an interview study poses several challenges (e.g., possible difficulties in recall, difficulties in understanding questions and interpreting verbal expressions), studies have shown that it is possible to actively involve this group in research (Novek & Wilkinson, 2019; Phillipson & Hammond, 2018). Therefore, this study investigated how residents with dementia experience a relocation from a regular nursing home to an innovative living arrangement.

## Methods

### Study design

This research was a qualitative study that used semi-structured interviews and observations based on an interpretative description approach; this approach aimed to capture the individual experiences of the residents and led to practical knowledge concerning relocating (Thompson Burdine et al., 2021; Thorne, 2016). To ensure transparency and rigor, the COREQ checklist was used for reporting. Also, this study was preregistered at onderzoekmetmensen.nl.

### Setting and participants

In this study, nursing homes were selected that offer 24 h care to older people with dementia with residents about to relocate from a traditional, large-scale nursing home to an innovative living arrangement. When the new location aimed to substantially change the physical, social, and/or organizational environment and presented itself as an alternative to regular nursing home care, it was regarded as an *innovative living arrangement*. Based on these criteria, two Dutch nursing homes were selected. Residents of these nursing homes were included when they (1) lived at the specified locations and received 24 h care there and (2) relocated from the old to the new location. All residents interviewed had dementia, and they were all care-dependent and in need of support in daily life.

In this study, a maximum variation sampling method was used, meaning we aimed to include a group of residents with a variety of demographic characteristics, such as gender and age (Moser & Korstjens, 2018). The sample size depended on data saturation, meaning that no new themes were observed in the data (Guest et al., 2006).

### Data collection

Our semi-structured interviews and observations occurred between June 2021 and May 2022. Interviews took place 2 weeks after the relocation in order to both prevent recall bias and increase the chance that residents had an active memory of the relocation process. A topic list was used as guidance, but interview questions were adjusted to the individual, meaning that when the participant struggled with answering questions, the questions were either rephrased or adjusted to enable participants to answer the question. The interviewers adapted the questions to the residents and how well they remembered the relocation. If the resident did not remember the relocation, the interviewers focused more on the new location and how this was experienced to gain insight in how the innovative environment was experienced.

The relocation consisted of three phases, including a pre-move phase, the actual relocation, and a post-move phase (Sussman & Dupuis, 2012). The topic list (see Additional File (1)) was based on the following phases: (1) weeks before the relocation (e.g., how did residents experience the weeks before the relocation?); (2) the relocation day (e.g., how did residents experience the relocation day?); (3) two weeks after the relocation (e.g., how did residents experience the first weeks after relocating to the innovative living arrangement?); and (4) the new location (e.g., how did residents experience the new location?).

In order to gain a deeper understanding of the experiences of residents, method triangulation was used by making observations. The researcher (MB) followed residents in the weeks prior to relocation, the relocation day, and the weeks after the relocation. Furthermore, the researcher attended preparatory meetings that focused on the relocation, such as family gatherings, vision meetings, project meetings, and other related meetings. Throughout all observations, informal conversations with residents, family members, and staff members took place and field notes were taken. These field notes described observations of the relocation process, the care environment, how the participants acted and reacted throughout the relocation process, and personal impressions concerning the atmosphere.

When care organizations agreed to participate, the legal representatives of the residents received information concerning the study and were asked to provide written informed consent for the resident’s participation. Furthermore, the participants were also asked to provide oral consent for participating in the interview. Interviews took place at the included locations and at the convenience of the residents. Demographic data concerning age, gender, and the care provided were collected.

### Data analysis

All interviews were transcribed verbatim and analysed with thematic analysis (Braun & Clarke, 2012). An inductive coding approach guided by the research questions was employed (de Casterlé et al., 2012; Dierckx de Casterlé et al., 2021). Initially, each interview was comprehensively read to get familiar with the content. Subsequently, summaries of the interviews were created to describe their core essence; the summaries were then validated by the participants through a member check. Next, the interviews were coded, in which all relevant text was assigned a corresponding ‘code’. Throughout the coding process, the codes remained as close to the text as possible. The qualitative data analysis software MAXQDA was used to facilitate this process (GmbH, 1989). The codes were subsequently organized into distinct themes. Throughout this iterative process, interviews were continually compared, codes refined, and emerged themes crosschecked against existing data. Two researchers (MB, EL) coded the interviews, and 10% of the interviews were coded independently by both and compared in order to ensure analytical rigor. Findings were then discussed within the research team. Field notes were examined using a narrative approach (McAdams, 2005), wherein the first author (MB) reviewed the field notes repeatedly to recognize overarching narratives, themes, characters, and events, aligning them with the interview data for further validation.

### Ethics

The Medical Ethics Committee of Zuyderland confirmed that the regulations under the Medical Research involving Human Subjects Act do not apply to this study (registration number: METCZ20210065). All legal representatives of the residents received information concerning the study and provided written consent.

## Results

### Sample characteristics

A total of 16 participants were included in this study. The interviews lasted an average of 19 min (range: 5–44 min). Residents were on average 86 years old (SD = 4.7; range: 77–93). Of the participants, eight were male and eight were female. The old location of the first nursing home was a large facility that contained two wards. Each ward had about 30 rooms, three long hallways, and two dining rooms. The wards consisted of about 30 residents each and the staff member pool was large, meaning no fixed staff team was appointed to the ward. When relocating, the residents moved to smaller houses in a park-like setting in the same village with the aim to enhance freedom of movement and provide more person-centred care. The old location of the second nursing home selected for this study was a building that resembled an apartment building. It consisted of five floors with about seven residents per floor, with one kitchen/dining room per floor. The residents moved to a location in another village that was inspired by aspects of green care farms (i.e., a facility that combines care and agricultural activities; (de Boer et al., 2017)). The aim of the new location was to focus on the strength and independence of residents and instead of doing activities for them, doing activities together.

First, the emotions and experiences of residents when relocating were summarized. Then, five themes emerged from the data, describing what was important for residents when moving (see Table 1 for a summary of findings). The identified themes showed that residents experienced several needs that were of importance throughout the entire relocation process. The themes pertained to (1) physical environment of the new location, (2) belongings of residents, (3) feeling at home, (4) the importance of social interaction when relocating, and (5) the need to be engaged in daily life.

**Table 1.** Summary of findings.

Theme	Residents’ perspective	Family/facility support
The physical environment of the new location	Most residents seemed satisfied with the new location. However, some residents were less enthusiastic, placing emphasis on how the location was decorated and wayfinding	The physical environment was designed by an external party. Residents were not actively involved in development and decoration of the new location
Belongings of residents	The belongings of residents were important. In the weeks before relocating, residents expressed fears of belongings getting lost or being stolen. The absence of belongings led to distress, but presence of their belongings led to increased calmness	Belongings were packed and unpacked by family, staff or the moving company. Residents did not pack belongings themselves
Feeling at home	Feeling at home was important for residents. Both at the old and new location, feeling at home differed between residents. Residents that did not feel at home, indicated not liking the decorations, that the new location felt strange, or wanting to leave	-
The importance of social contact when relocating	Residents appreciated social contact of both family and staff. Staff were often mentioned in a care related context, except during the relocation day	Family members joined during the relocation day and visited their relatives after the relocation. Staff mostly provided care related support, but their role changed in providing social support during the relocation day
The need to be engaged in daily life	Residents wanted to be of use and involved. They wanted to spend their daily lives in a meaningful way, and as independent as possible	Residents were not involved in decision making concerning the new environment and the relocation process. Furthermore, they received less information concerning the relocation than other stakeholders, such as their relatives

### The emotions and experiences of residents when relocating

The residents who recollected the actual relocation described this experience either as intense or as normal, or positive in hindsight. This corresponded with the observations of the researcher and was visible in the weeks before the relocation and the relocation day itself. Residents expressed mixed feelings, ranging from feelings of stress and agitation, to feeling calm throughout this process. During the relocation day, some residents remained seated in the area where they waited for their belongings to be moved. They played games, listened to music, or talked with one another. Overall, they expressed feelings of calmness and patience. Other residents, however, walked around looking for exits and expressed agitation:

(field note, relocation day) [“One resident shows signs of discomfort and agitation. When staff members approach her, she tells them that she does not like this one bit and wants to leave”.]

When first arriving at the new location, most residents seemed to be overwhelmed. They sat in the kitchen in chairs and seemed to withdraw, not being directly able to emotionally respond to the new location and all the stimuli they encountered. Staff members later stated that this withdrawing behaviour in most residents lasted 1 day.

### The physical environment of the new location

For residents it seemed easiest to talk about the physical environment of the new location. It seemed to be tangible to them, as they could talk about something they could directly see and feel. Although residents were clearly able to talk about the physical environment and what they either liked or did not like, the physical environment was designed by an external party, meaning that external outside and inside architects were approached to design the building, but also the inside interior. Residents were therefore not actively involved in the development and decoration of the new location. Most residents seemed to be satisfied with the new location, however. Residents liked the new location, stating it was nice, larger, and beautiful. When asking the residents how they liked the new location, one of the residents responded:

(resident, 91, interview 03_03) [“Good. Yes, I have a room to myself here so, um. Everything is here to be able to sleep. (…) I like that there is enough space”.]

Most residents also responded positively when they first entered the new location, which they usually saw for the first time during the relocation day, stating that it was beautiful. Some residents were triggered to immediately explore the new location and look around. When talking about the facilities, residents mentioned several things. For example, they liked the restaurant, the food, the bathroom, or how clean the building was. Some residents, however, were less enthusiastic about the new location, placing particular emphasis on how the location was decorated. One resident emphasized that the location looked “too modern” and was missing decorations. The resident missed the old paintings and furniture in the common and private rooms that were always present in his previous homes:

(resident, 79, interview 03_02) [“I would hang more paintings. Fill it up a bit, as it is too empty now. Not three paintings, but six”.]

The way residents were able to find their way in the new building also differed. Some residents felt it was easy to find the way in the new building. Other residents, however, struggled with wayfinding, stating that the new hallways were confusing. One resident seemed to be very confused and lost all orientation in the new building. He felt like the environment was highly repetitive, and all the corners, doors, and colours looked alike.

### Belongings of residents

The belongings of residents were important to them. In the weeks before the relocation, residents often expressed the fear of belongings getting lost or being stolen. During the relocation day, multiple residents approached the researcher or staff members, stating that their belongings were stolen. Their belongings were either packed by family, staff members, or the moving company hired for the relocation day. Therefore, residents did not pack any belongings themselves. For some residents, certain items seemed to be essential, especially during stressful events (e.g., the relocation day), the weeks before relocating (e.g., when boxes were packed), or in the weeks after relocating (e.g., when everything was new, and routines were disrupted):

(field note, relocation day) [“I see one resident who is sitting between all the boxes that are already packed. She is waiting for staff members to take her to the restaurant for breakfast. She seems to be a bit agitated and actively clinches her purse to her chest. She keeps an eye on this purse throughout the entire day”.]

One resident owned a parrot, and she needed to have this parrot close to her in order to remain calm. When trying to separate her from her parrot to move the parrot during the relocation day, this resulted in a lot of agitation, leading to the decision to let her take the parrot with her. When arriving at the new location, the belongings were already in place, which comforted most residents. Some residents, however, seemed a bit displaced because the furniture was arranged differently, some belongings were still boxed, or they felt like some belongings were still missing.

### Feeling at home

For most residents, feeling at home was considered important. At the old location, residents did express feelings of either feeling at home or not. One resident, for example, often asked the researcher (MB) whether she wanted to see her room, which she had decorated entirely to her taste. She displayed clear signs of being proud of her room and surroundings. Another resident showed opposite behaviour, constantly looking for an exit and asking the researcher whether she could help him get back to his own house.

During the relocation day, residents often had to wait in a larger hall so their belongings could be transferred from the old location to the new one. During this day, many residents expressed a desire to go back to their own room in the old location. They were confused when they were told that this was not possible due to the relocation. They referred to the old nursing home as “home” or “my room”, indicating that they did associate the old location with home. One resident seemed to experience a sense of resignation from accepting that she was in a nursing home. When the interviewer asked how the resident felt about relocating to another house, the resident seemed to accept her situation, telling the interviewer that she had “come to terms” with her circumstances:

(resident, 85, interview 12_03) [“I have accepted the situation: I’ve been helped. It had to happen this way. That is what I think. (…) I’ve come to terms with it”.]

Residents who reported they felt at home indicated that it felt like home immediately after the relocation and that they did not even have to get used to their new environment. The residents who said they did not feel at home, however, indicated that they did not like the decoration of the location, the new location felt strange, or they wanted to go to their original homes where they still lived independently:

(resident, 79, interview 03_02) [“I like upholstered furniture, like the way it used to be, I’m used to that (…) and now I don’t have that anymore. I’ve worked for this for years and then you get transferred to another ward, you cannot go back to your own home. (…) No, I do not feel at home here”.]

Some of the residents recognized the village the new building was situated in, as it was the village they had lived in for most of their lives. Residents who recognized the village from their past seemed enthusiastic about being back in their “hometown”. Residents that recognized the village' name were calmer throughout the relocation process because they often felt happy going “back home”, and because it felt known and familiar.

### The importance of social contact when relocating

Most residents also mentioned the importance of having social contact throughout the relocation process. Residents described the importance of having social contact with staff members, family members, and/or fellow residents. When talking about the staff, most residents indicated they were satisfied with the way staff members were helping them with care-related tasks. They often referred to staff members as individuals who help and support residents. They were almost never referred to in a social context, meaning that the interactions residents had with staff were mostly care-related. This was in line with what the researcher observed in the weeks before the relocation and the weeks after the relocation. Staff members did engage in social contact with residents, but mostly with a care-related reason (e.g., asking them if they wanted coffee, asking them to take medication). However, during the relocation day, the social contact that residents had with staff members seemed more of a comforting nature, paying attention to their needs during the relocation day. When residents felt upset, they searched for comfort by approaching staff members, and staff members actively comforted these residents. It was evident that residents needed this support throughout the day. The residents who did not display agitation were actively involved in small talk or entertained by playing games or listening to music together. Music appeared to be a strong binding factor between residents and staff. Throughout all observation moments, music was often used as topic of conversation, a way of comforting residents, or as an activity of singing together. When discussing family members, residents almost always referred to them in a social context, indicating they enjoyed having family over in general, like going out to a restaurant together, for example. Some residents mentioned that family members helped in the relocation process, either by physically relocating belongings, or emotionally, by helping the resident get accustomed to the new location:

(resident, 89, interview 04_03) [“Yes, I had to get used to it, of course. But there were people who wanted to visit me. I found that quite nice”.]

When talking about fellow residents, some indicated they could get along well with the fellow residents; others either indicated not having a lot of contact with fellow residents or not being able to talk properly to them. In the latter case, residents noticed that the fellow residents acted “differently”. For example, residents who were in an earlier stage of dementia acknowledged that fellow residents had dementia or described them as hard to connect with:

(resident, 89, interview 05_03) [“That is what happens with dementia – suddenly you do not recognize people anymore. You have to understand that, and you cannot judge, either. You cannot judge the people who are there. You cannot; they have dementia. (…) You have to accept that and remain reasonable and think: ‘Yes, glad that has not affected me yet’“.]

### The need to be engaged in daily life

Residents showed signs of wanting to be of use and involved, but despite this fact, they were not actively involved throughout the relocation process. They did not have a say in how their new environment would look like; they were not involved in the packing or other preparatory aspects; and they received less information concerning the relocation than, for example, their relatives. Residents expressed a desire to remain engaged and relevant. When talking to the researcher, residents often talked about their jobs or households and how hard they had to work. It was important for them to emphasize that they were independent, able to earn enough money, keep their houses clean and proper, and raise their family. Multiple residents, for example, expressed this also by their behaviour, by actively helping staff members in household chores or by taking care of fellow residents:

(field note) [“One resident is helping her fellow resident. She slices the crust of the bread and asks the fellow resident whether she would like something to drink”.]

Administrators at both locations had the intention to provide a larger and more interactive outside area in order to create more opportunities to be outside and engage in outside activities. However, despite the clear desire residents expressed in being outside and active, no large differences in the amount of time spent outside was observed despite the fact that most residents stressed the importance of staying active and engaged during the day, being outside, and being able to do what one wants to do. Some residents liked music, others liked helping the staff with household tasks, but in general, when asked what residents liked to do during the day, “walking outside” was mentioned the most. There seemed to be a need to be outside and a need to maintain physical activity:

(resident, a85, interview 12_03) [“He (staff member) had to put on the jacket. ‘Don’t you have a jacket?’ he asks. I say, ‘Yes.’ ‘Come’, he says, ‘then let’s go’. Then he takes a wheelchair, and he places me in it. And then he goes for a walk with me. (…) That’s something I need to have”.]

## Discussion

In this study we aimed to investigate how residents with dementia experienced a relocation from a regular nursing home to an innovative living arrangement. Overall, we found that although residents showed clear needs concerning the relocation process, they were not actively involved. When residents described the emotions they experienced throughout the relocation process, these ranged from negative to positive. Furthermore, certain themes appeared to be of importance to the residents, including the physical environment of the new location; their belongings; whether they felt at home at the new location; the social contact residents had with their environment when relocating; and the need to be engaged in daily life.

Innovative living arrangements are designed to prioritize values like autonomy, freedom of choice, independence by normalizing daily life (e.g., forming a household with residents and staff) and offering choice (Brouwers et al., 2023). However, these values were not obviously reflected in this study’s relocation process, as residents were not involved in decision-making throughout the relocation process. When considering the two cases included in this study, the building and interior were all designed by external parties and residents did not have a say in the creation of the physical environment or in how the relocation process (e.g., packing bags, the relocation day) was planned. Research shows that involving residents in the relocation process can lead to more positive outcomes (Bekhet et al., 2011; Jolley et al., 2011; Leyland et al., 2016; Weaver et al., 2020). Furthermore, autonomy has been considered important for residents and might have an influence on the experienced quality of life (Caspari et al., 2018; McCabe et al., 2021). Relationships between staff and residents are very important and can either inhibit or promote autonomy in residents with dementia, however (van Loon et al., 2021). Thus, staff have a crucial role to play in promoting autonomy during relocations.

Involving residents, however, is not necessarily easy, as struggles between offering freedom and ensuring safety can arise. Thus, withholding information is a frequently used strategy within nursing home care for residents with dementia in order to reduce anxiety, stress, or other negative emotions (Tuckett, 2012). Previous studies have shown that although residents with dementia value participation in decision-making, they often lack the opportunity (Daly et al., 2018). In line with the literature, both included care organizations in this study chose the tactic of organizing the relocation without the active involvement of residents to minimize relocation stress. However, a relocation is stressful for staff as well, and some research showed that how staff acts and feels impacts the well-being of residents and possibly causes stress for residents either way (Anderson et al., 2016). Therefore, the strategy of withholding information and not involving residents might not be as effective as expected.

The residents stressed the importance of feeling at home. Research shows that the subjective experience of residents and the innovative properties of the new environment shape their overall well-being and adjustment following relocation (Bekhet et al., 2011; Nowlan et al., 2015), and especially the presence of a ‘homey’ feeling is important (Ibrahim et al., 2022). Some research has shown that social and psychological aspects like autonomy, self-identity, control, choice, and a socially supportive environment are necessary to create feelings of home (Cutchin et al., 2003; Moore, 2000). In order to be able to create a place that enables at-homeness, knowing what matters to an individual and helping residents feel like they still matter is key (Cater et al., 2022; Molony et al., 2011; van Hoof et al., 2016). Within innovative living arrangements, fostering ‘a sense of home’ is often central to the care philosophy, emphasizing individualized approaches that prioritize residents’ preferences, routines, and social interactions (Brouwers et al., 2023). Past research on the impacts of or improvement of relocations within long-term care mainly focused on physical and mental health outcomes (e.g., depression) of residents, thereby neglecting the importance of feeling at home (Broekharst et al., 2022; Weaver et al., 2020) despite the clear desire that residents generally show in needing to feel at home. More emphasis might be placed on how to create a home when relocating to a new location instead of only attempting to diminish the negative emotions of residents (and staff) throughout the relocation process.

## Implications for research and practice

This study showed that residents with dementia can communicate their needs and wishes concerning relocation processes. They did not emphasize matters specifically related to the upcoming innovation and culture change, but instead focused on overall, more general needs. This implies that it is important to particularly emphasize these overall needs when relocating towards an innovative living arrangement. Healthcare professionals might optimize relocation processes by actively involving residents and adhering to the needs and wishes they express. First, healthcare professionals should place more emphasis on shared decision-making, by including residents throughout the relocation process. Furthermore, they could create a sense of home, for instance by packing boxes together and involving residents in the design and decoration of new locations. Third, gaining insight into the individual preferences and wishes of residents is important for optimizing the relocation process. As most of the related interventions have focused mostly on the preparatory phase, more emphasis should be placed on the phase after relocation, where the residents and staff might work together on creating a feeling of home.

Actively involving residents with dementia, however, can be seen as a challenge by staff. Therefore, future research should explore how to optimally involve residents and provide tools and aids for staff members and other stakeholders. Furthermore, we encourage future researchers to explore best practices concerning the planning of the relocation phase, and the needs and wishes of residents should ideally be represented in these best practices.

## Methodological considerations

In order to capture the ‘lived experiences’ of the relocation process, we decided to interview the residents 2 weeks after the actual relocation day. Therefore, the interviews were planned as close as possible to the phenomenon of interest. However, planning the interviews later in time and observing the residents longitudinally might have led to additional useful insights, like additional insights into how residents were supported in getting used to their new environment and whether their needs and preferences changed over time. Although the participants who suffered from dementia often provided short answers (e.g., due to recall issues or difficulties with speech), we were able to capture the experiences of residents. Finally, due to our employment of the method of triangulation, more context was provided through observations and informal conversations.

## Conclusion

With this study we showed that it is important to involve residents throughout the relocation process, as residents with dementia were able to express what was important to them. Both healthcare professionals and policy officers should actively involve residents with dementia throughout the relocation process. Instead of only diminishing negative emotions, the relocation process should focus also on increasing the involvement of residents in order to be able to increase the residents’ autonomy and help the residents continue to feel at home.

## Supplemental Material

Supplemental Material - Experiences and needs of residents with dementia in relocating to an innovative living arrangement within long-term care: A qualitative study Supplemental Material for Experiences and needs of residents with dementia in relocating to an innovative living arrangement within long-term care: A qualitative study by Mara Brouwers, Elleke GM Landeweer, Bram de Boer, Wim G Groen, Miranda C Schreuder, and Hilde Verbeek, on behalf of the RELOCARE consortium in Dementia: the international journal of social research and practice
