# Supplementary material for: Experiences and needs of residents with dementia in relocating to an innovative living arrangement within long-term care: A qualitative study
Source: Dementia (London). 2024 Dec 21;24(7):1225–40. doi: 10.1177/14713012241311433 (PMC12408925; doi:10.1177/14713012241311433)
Supplement: Supplemental Material - Experiences and needs of residents with dementia in relocating to an innovative living arrangement within long-term care: A qualitative study [file sj-pdf-1-dem-10.1177_14713012241311433.pdf]

Ervaring omtrent verhuizing: Bewoner

<table>
<tr><td colspan="3">Ervaringen van bewoner rondom de verhuizing</td></tr>
<tr><td colspan="3">Inleiding interview: Ik wil het graag met u hebben over de verhuizing die heeft plaatsgevonden. Ik ben vooral geïnteresseerd in hoe u deze verhuizing heeft ervaren en wat u als positief en negatief hebt ervaren tijdens het verhuizen. Hier zullen de meeste vragen dan ook over gaan. Graag zou ik willen beginnen met een brede algemene vraag: Hoe bevalt uw nieuwe huis?</td></tr>
<tr><td>Hoofdvraag</td><td>Interviewvragen</td><td>Categorie</td></tr>
<tr><td rowspan="5">Hoe heeft de bewoner het verhuizen van of naar een innovatief woonzorgconcept ervaren?

Welke aspecten heeft hij/zij als negatief ervaren en welke als positief?

Hoe ziet het sociale netwerk van de bewoner eruit?</td><td>Hoe bevalt uw nieuwe huis?</td><td>Initiële vraag</td></tr>
<tr><td>Hoe is de aanloop naar de verhuizing verlopen?
Wat vond u van de oude locatie?
Wat de reden van uw verhuizing? Is de verhuizing vrijwillig of niet?
Op welke wijze werd u voorbereid op de verhuizing?
Wanneer werd u geïnformeerd over de verhuizing?
Wat vond u van het nieuws dat u ging verhuizen?
Heeft u de nieuwe locatie voor de verhuizing gezien en/of bezocht?
Zo ja, hoe heeft u dit bezoek ervaren?
Hoe heeft u de weken voor het verhuizen ervaren?</td><td>Aanloop verhuizing</td></tr>
<tr><td>Hoe is de verhuizing zelf verlopen?
Zou u me stap voor stap uit kunnen leggen hoe de verhuizing is verlopen? (Bijvoorbeeld, hoe zijn spullen verhuist, welke wijze van vervoer, samen met andere bewoners of alleen verhuist etcetera)
Vond u de verhuizing goed georganiseerd?
Zo ja, waarom wel?
Zo nee, waarom niet?
Hoe heeft u de verhuizing ervaren?</td><td>Verhuizing</td></tr>
<tr><td>Hoe is het gewennen aan uw nieuwe huis verlopen?
Hoe heeft u de eerste week na de verhuizing ervaren?
Op welke wijze werd u geholpen aan de nieuwe omgeving te wennen?</td><td>Nasleep verhuizing</td></tr>
<tr><td>Hoe bevalt de nieuwe omgeving?
Hoe bevalt de nieuwe indeling van de gebouwen?
Hoe bevalt de nieuwe werkwijze van het personeel?
Hoe bevallen de nieuwe faciliteiten?</td><td>Nieuwe locatie</td></tr>
</table>
